# DACH1 suppresses breast cancer as a negative regulator of CD44

**DOI:** 10.1038/s41598-017-04709-2

**Published:** 2017-06-28

**Authors:** Hanxiao Xu, Shengnan Yu, Xun Yuan, Jing Xiong, Dong Kuang, Richard G. Pestell, Kongming Wu

**Affiliations:** 1 Department of Oncology, Tongji Hospital of Tongji Medical College, Huazhong University of Science and Technology, Wuhan, 430030 China; 2 Department of Pathology, Tongji Hospital of Tongji Medical College, Huazhong University of Science and Technology, Wuhan, 430030 China; 3 Department of Cancer Biology, Sidney Kimmel Cancer Center, Thomas Jefferson University, Philadelphia, PA 19107 USA

## Abstract

Dachshund homolog 1 (DACH1), a key cell fate determination factor, contributes to tumorigenesis, invasion, metastasis of human breast neoplasm. However, the exact molecular mechanisms for the anti-tumor roles of DACH1 in breast carcinoma are still lack of extensive understanding. Herein, we utilized immunohistochemistry (IHC) staining and public microarray data analysis showing that DACH1 was higher in normal breast, low-grade and luminal-type cancer in comparison with breast carcinoma, high-grade and basal-like tumors respectively. Additionally, both correlation analysis of public databases of human breast carcinoma and IHC analysis of mice xenograft tumors demonstrated that DACH1 inversely related to cancer stem cells (CSCs) markers, epithelial-mesenchymal transition (EMT) inducers and basal-enriched molecules, while cluster of differentiation 44 (CD44) behaved in an opposite manner. Furthermore, mice transplanted tumor model indicated that breast cancer cells Met-1 with up-regulation of DACH1 were endowed with remarkably reduced potential of tumorigenesis. Importantly, meta-analysis of 19 Gene Expression Omnibus (GEO) databases of breast cancer implicated that patients with higher *DACH1* expression had prolonged time to death, recurrence and metastasis, while *CD44* was a promising biomarker predicting worse overall survival (OS) and metastasis-free survival (MFS). Collectively, our study indicated that CD44 might be a novel target of DACH1 in breast carcinoma.

## Introduction

In spite of significant achievement made in early diagnosis and therapeutic strategies, breast cancer still draws great attention from the worldwide because of its high incidence rate and mortality^1–3^. The unsatisfactory clinical outcome is mostly due to tumor recurrence, metastasis and therapy-resistance^1^. Identifying novel biomarkers related to molecular subtypes, aggressive phenotypes and prognosis of breast cancer is essential for drug development, disease surveillance and precise therapy.

The retinal determination gene network (RDGN), including DACH1, EYA1 and SIX1, plays crucial roles in the development of multiple organs^4^. SIX1 and EYA1, two important RDGN members, exert favorable effects on tumor initiation and progression^4, 5^, and high expression of SIX1 and EYA1 is an adverse factor for clinical outcomes for breast cancer patients^6–8^. On the contrary, another key RDGN member DACH1 behaved as a tumor suppressor and reduced expression of DACH1 predicts poor survival performance of breast cancer patients^9^. Several lines of evidence have demonstrated that the hypermethylation of promoter region leads to the down-regulation of DACH1, which is closely associated with proliferation, invasion and metastasis of various tumors, including breast cancer^10–13^, lung cancer^14^, esophageal cancer^15^, renal cell carcinoma^16^ and hepatocellular carcinoma^17^. DACH1 antagonizes the transcription and translation of oncogenes and induces epithelial-mesenchymal transition (EMT) in breast cancer, resulting in the inhibition of tumor growth, invasion and migration^9, 10^. Recent studies prove that cancer stem cells (CSCs) possess potent self-renewal ability and are responsible for tumor relapse and metastasis and endogenous DACH1 participates in the negative regulation of CSCs^18, 19^.

Cluster of differentiation-44 (CD44), a ubiquitously present glycoprotein on the membrane of mammalian cells, plays essential roles in a variety of biological function such as cell division, adhesion and migration^20^. During the past decades, the role of CD44 in cancer development has been revealed and valued. As a well-known marker of CSCs, CD44 promotes carcinogenesis, invasion, metastasis and therapy-resistance^20–23^. It promotes proliferation and suppresses apoptosis by regulation of relative pathways, including Ras-Raf-Mek-Erk-Cyclin D1 pathway and phosphoinositide 3-kinase (PI3K)-Akt signaling, as well as stimulates EMT, which contributes to tumor invasion and metastasis^20, 24^.

Previous study has demonstrated that endogenous reduction of DACH1 was accompanied by down-regulation of CSC markers, such as SOX2, Nanog, KLF4^18^. To further evaluate the correlation between DACH1 and CSC markers and EMT inducers in breast cancer, we performed a comprehensive analysis of immunohistochemistry (IHC) staining, publicly available microarray data, RNA profiling and western blot. Our study indicated that DACH1 was inversely correlated with CD44 and CD44 might be a novel target of DACH1 in breast cancer.

## Results

### DACH1 and CD44 associated with tumorigenesis and histological grade of breast cancer

**Figure 1:**
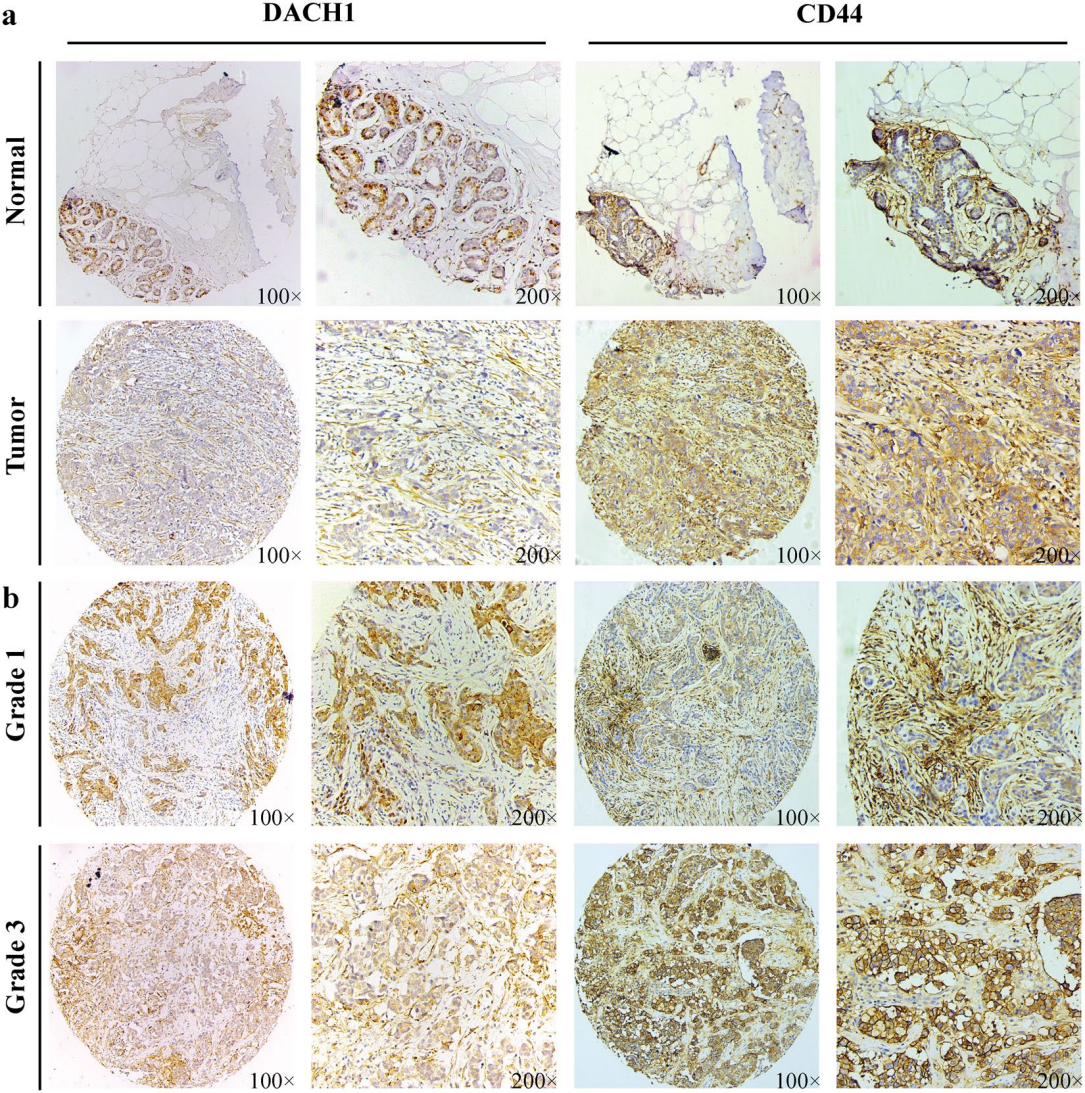
DACH1 and CD44 were correlated with tumorigenesis and histological grade of breast carcinoma. (**a**) Representative images of immunohistochemistry staining of DACH1 and CD44 in noncancerous and cancerous tissues were shown. (**b**) Representative images of immunohistochemistry staining of DACH1 and CD44 in low-grade and high-grade breast neoplasm tissues were shown.

In order to evaluate the expression of DACH1 and CD44 in normal breast and breast malignant tissues, we carried out IHC analysis on two TMAs (BR1502–97 and BR1502-98) with normal breast and human breast cancer tissues. DACH1 was majorly found in nucleus and CD44 was mostly detected on the membrane of breast cancer cells. Representative images of IHC staining for noncancerous and cancerous tissues were shown in Fig. 1a, showing that DACH1 decreased and CD44 increased in breast neoplasm tissues in comparison with normal breast.

Additionally, we also explored the correlation between the protein abundance of DACH1 and CD44 and histological grade. Representative images of IHC staining for low-grade and high-grade cancerous tissues were showed in Fig. 1b, which indicating that DACH1 was inversely correlated with tumor grade, while CD44 was positively associated with histological grade.

### Expression of DACH1 and CD44 correlated with molecular subtypes of breast cancer

**Figure 2:**
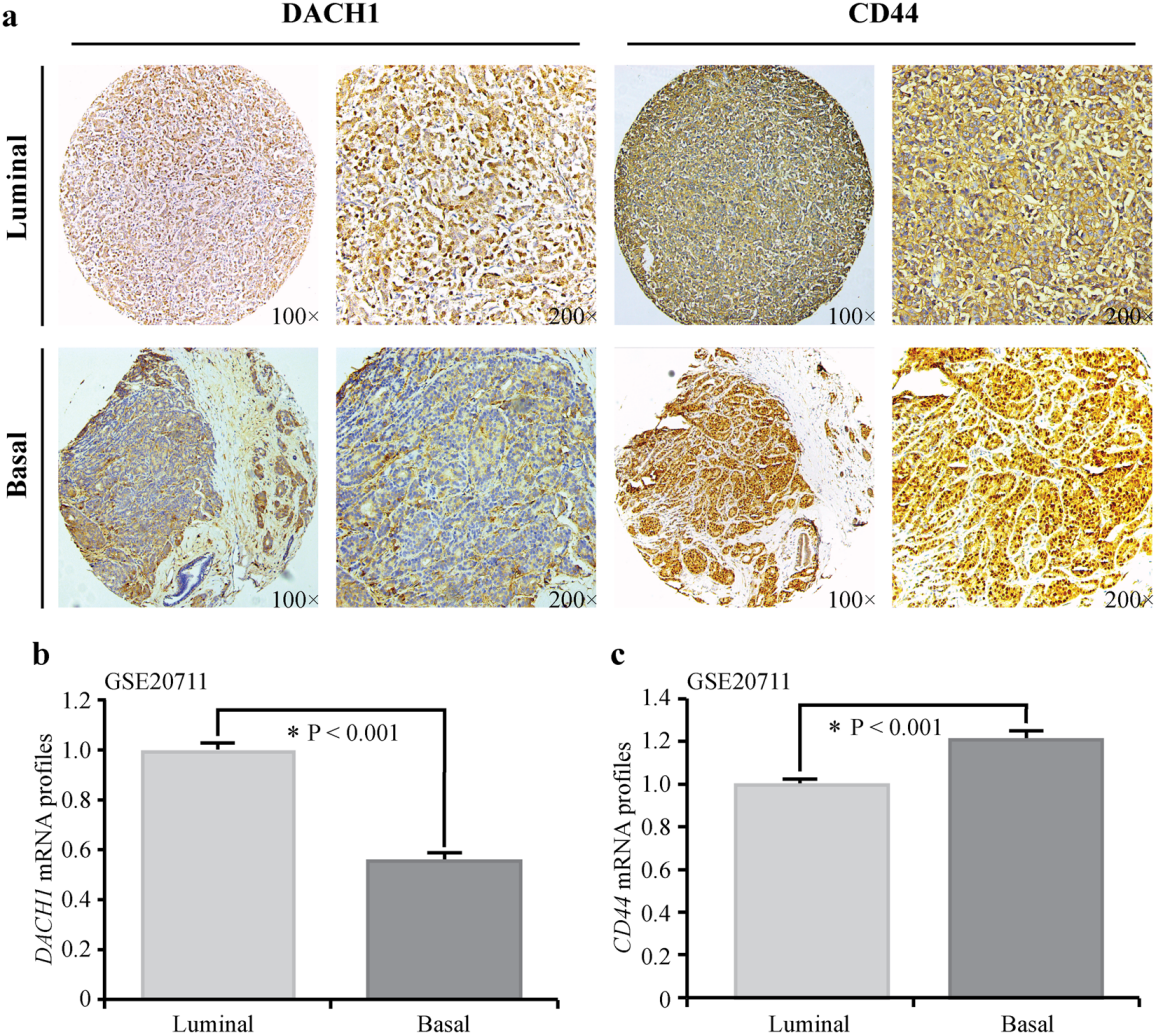
The expression of DACH1 and CD44 correlated with molecular subtypes of breast carcinoma. (**a**) Representative images of immunohistochemistry staining of DACH1 and CD44 in luminal-type and basal-like breast cancer tissues were shown. (**b**) Expression analysis of public microarray dataset GSE20711 showed that the mRNA level of *DACH1* was significantly higher in luminal-type than in basal-like breast tumor. (**c**) Expression analysis of GSE 20711 also displayed that *CD44* mRNA expression was remarkably lower in luminal-type than in basal-like breast cancer.

We also carried out IHC staining to assess the protein abundance of DACH1 and CD44 in luminal-type and basal-like breast cancer tissues. Representative images for the expression of DACH1 and CD44 in luminal and basal tissues were shown in Fig. 2a. Furthermore, expression analysis of GSE20711 including a total of 45 luminal and 22 basal-like breast tumor cases was also interrogated to evaluate the mRNA levels of *DACH1* and *CD44* in luminal and basal-like breast neoplasm tissues, which showed that *DACH1* was enriched in luminal breast carcinoma in comparison with basal-like breast cancer (P < 0.001) (Fig. 2b), while *CD44* exhibited an opposite tendency (P < 0.001) (Fig. 2c) at mRNA level. Altogether, our results indicated that luminal breast carcinoma was most likely to be DACH1^high^/CD44^low^ type, and basal-like breast tumor tissues were majorly DACH1^low^/CD44^high^ type.

### DACH1 down-regulated some CSC and EMT markers *in vitro*, as well as blocked Met-1 tumor growth *in vivo*

**Figure 3:**
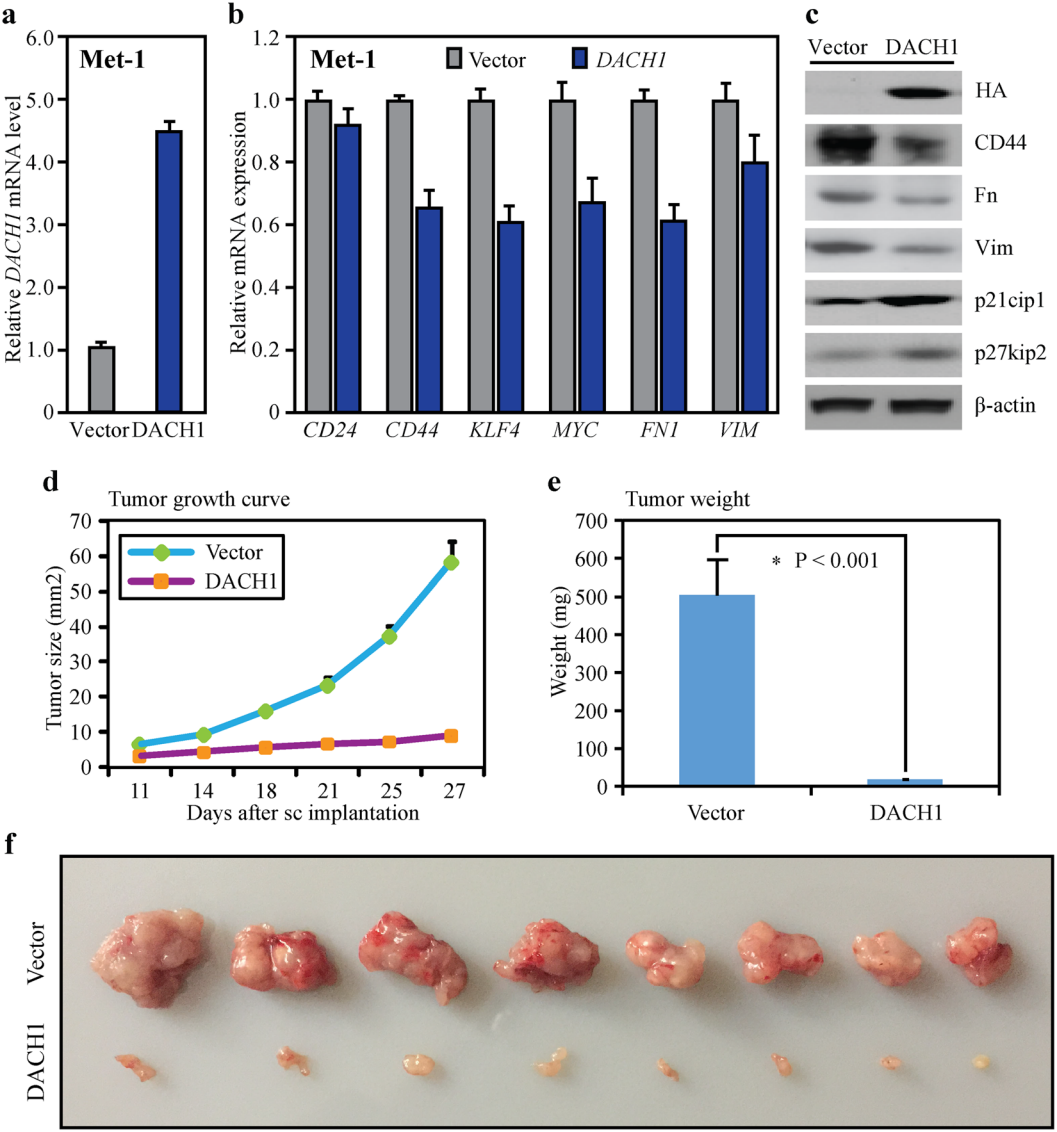
DACH1 regulated the expression of some CSCs and EMT genes in Met-1 cells and suppressed tumor growth *in vivo*. (**a**) Stable expression of *DACH1* in breast cancer Met-1 cells was achieved by retrovirus infection. (**b**) RNA microarray and cluster analysis showed that upregulation of *DACH1* reduced the mRNA levels of *CD24*, *CD44*, *KLF4*, *MYC*, *FN1* and *VIM* in Met-1 cells. (**c**) Western blot indicated that upregulation of DACH1 reduced the protein abundance of CD44, Fibronectin, Vimentin, p21 and p27 in Met-1 cells. (**d**) DACH1 overexpression significantly reduced the volume of tumors by ∼90% and slowed down the speed of tumor growth in nude mice xenograft tumors. (**e**) Overexpression of DACH1 also reduced mice transplanted tumor weight by ∼90%. (**f**) Mammary tumor growth *in vivo* was evaluated by subcutaneous implantation of DACH1-overexpressing Met-1 cells and the GFP controls in nude mice.

Breast cancer Met-1 cells were transducted with a *DACH1* expression vector resulting in an ∼4.5-fold increase in *DACH1* expression (Fig. 3a) and subsequent reduction of CSC markers *CD44*, *KLF4* and *MYC* as well as EMT markers including *FN1* and *VIM* (Fig. 3b) by mRNA analysis. Western blot also demonstrated the presence of the DACH1-tagged FLAG epitome, and the effects of DACH1 overexpression on the protein abundance of CD44, Fibronectin, Vimentin, p21 and p27, which were showed in Fig. 3c. Ectopic expression of DACH1 contributed to remarkable reduction of CD44, Fibronectin, Vimentin and significant up-regulation of p21 and p27 in Met-1 cells. Mammary tumor growth *in vivo* was assessed by subcutaneous implantation of Met-1 cells in nude mice (Fig. 3f). Met-1 cells with engineered expression of DACH1 were endowed with remarkably reduced potential of tumorigenesis in xenograft tumors. Up-regulation of DACH1 significantly reduced the volume of tumors by ∼90% and slowed down tumor growth (Fig. 3d). Tumor weight was also reduced by ∼90% in comparison with the control tumors (Fig. 3e). The results implicated that DACH1 suppressed the expression of some CSCs and EMT markers and serves as a potent anti-tumor factor in xenograft tumors.

### DACH1 reduced the expression of CD44, Fibronectin, Vimentin, Myc, Sox2, EGFR, Ki-67 *in vivo*

**Figure 4:**
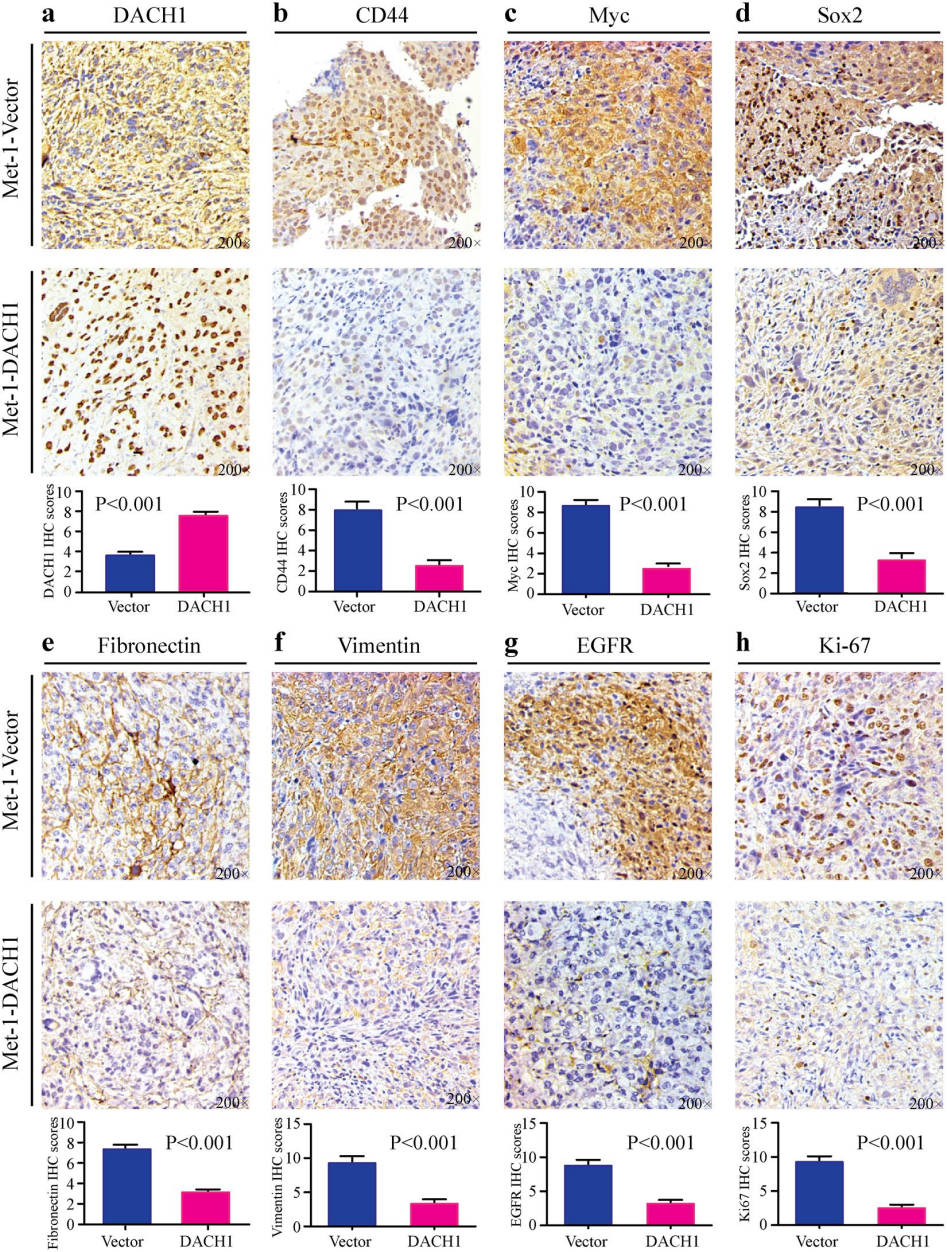
DACH1 reduced the expression of CD44, Myc, Sox2, Fibronectin, Vimentin, EGFR and Ki-67 *in vivo*. Both representative immunohistochemistry images and scoring results showed that overexpression of DACH1 (**a**) down-regulated the protein abundance of CD44 (**b**), Myc (**c**), Sox2 (**d**), Fibronectin (**e**), Vimentin (**f**), EGFR (**g**) and Ki-67 (**h**) in nude mice xenograft tumors.

Immunohistochemistry analysis was conducted to assess the protein abundance of DACH1, CD44, Myc, Sox2, Fibronectin, Vimentin, EGFR, Ki-67 in nude mice xenograft tumor tissues with overexpression of DACH1 and the GFP controls. Additionally, we also employed IHC scoring to quantize the levels of these proteins in both DACH-overexpressing and the control tumors by using semi-quantitative criteria. About six 200 magnification images of each kind of protein were selected for IHC scoring by two experienced pathologists independently. Representative images of IHC staining and scoring results for DACH1, CD44, Myc, Sox2, Fibronectin, Vimentin, EGFR and Ki-67 were shown in Fig. 4a,b,c,d,e,f,g and h, respectively. Our results displayed that over-expression of DACH1 (P < 0.001) remarkably reduced the expression of CD44 (P < 0.001), Myc (P < 0.001), Sox2 (P < 0.001), Fibronectin (P < 0.001), Vimentin (P < 0.001), EGFR (P < 0.001) and Ki-67 (P < 0.001), demonstrating that DACH1 could potently down-regulated the expression of some CSCs and EMT markers, basal-like factor EGFR and proliferative biomarker Ki-67 *in vivo* at protein level.

### Correlation between the expression of *DACH1* and *CD44* and the levels of *FN1*, *VIM*, *YBX1*, *FOXA1*, *EGFR* and *MKI67*

**Figure 5:**
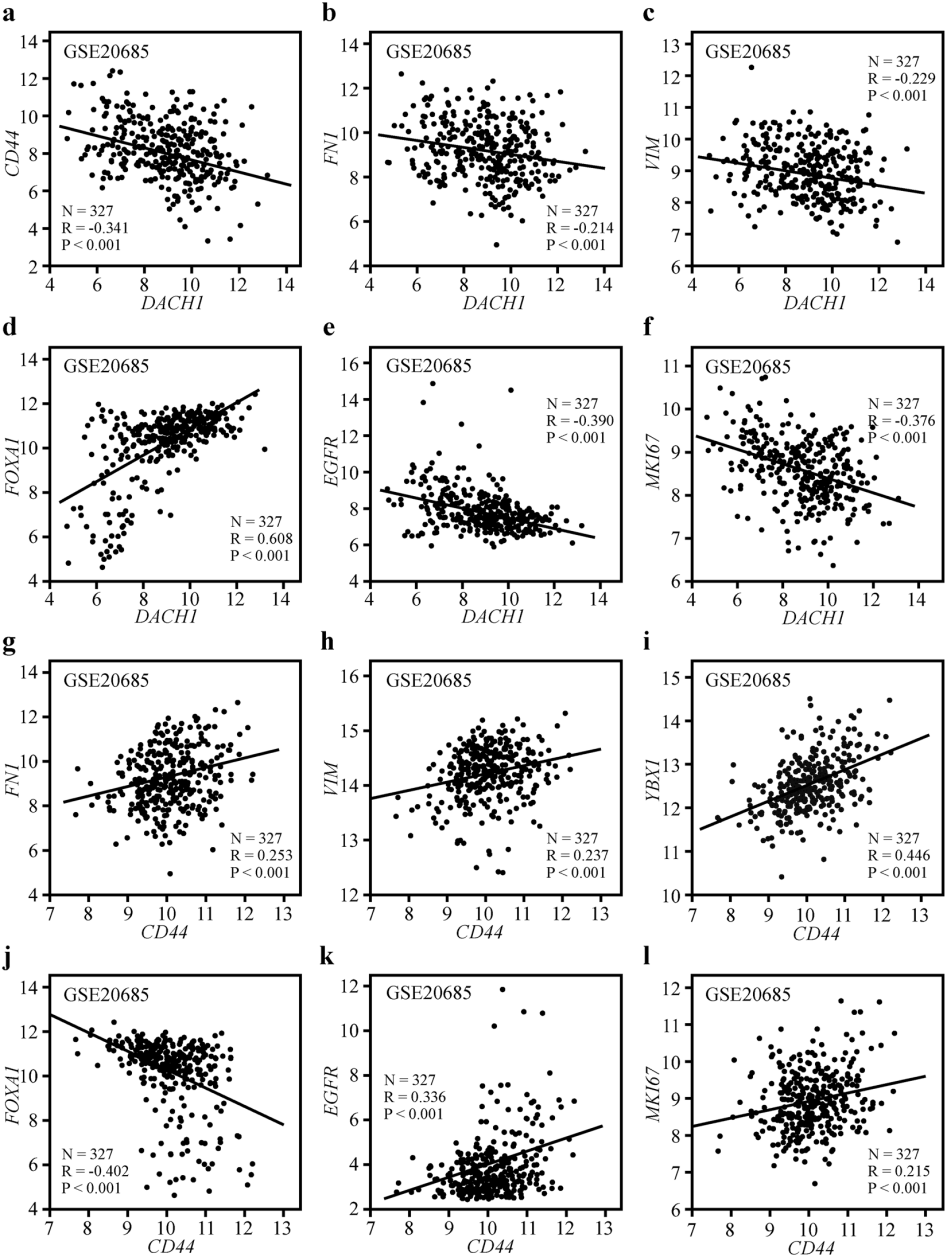
The expression of *DACH1* and *CD44* correlated with *VIM*, *FN1*, *YBX1*, *FOXA1*, *EGFR* and *MKI67* in breast cancer tissues. Correlation analysis of public dataset GSE 20685 showed that *DACH1* was inversely correlated with cancer stem cell marker *CD44* (**a**), mesenchymal markers *FN1* (**b**) and *VIM* (**c**) as well as basal-like markers *EGFR* (**e**) and *MKI67* (**f**), while positively associated with luminal marker *FOXA1* (**d**). *CD44* was parallel with *FN1* (**g**) and *VIM* (**h**), *YBX1* (**i**), *EGFR* (**k**) and *MKI67* (**l**), while negatively associated with *FOXA1* (**j**).

Previous study has implicated that DACH1 enriched in luminal A breast cancer and its expression fluctuated in direct proportion to the level of luminal-like marker FOXA1^13^. Previously experimental study demonstrated that DACH1 participated in the inhibition of Snail-induced EMT through suppressing the activity of the Y box-binding protein (YB-1)^9^. Herein, public dataset GSE20685 was interrogated to assess the association between *DACH1* and *CD44*, *FN1*, *VIM*, *FOXA1*, *EGFR* and *MKI67*, as well as evaluate the correlation between *CD44* and the above genes. The results showed that *DACH1* mRNA expression was inversely correlated with *CD44* (R = −0.341, P < 0.001) (Fig. 5a), *FN1* (R = −0.214, P < 0.001) (Fig. 5b), *VIM* (R = −0.229, P < 0.001) (Fig. 5c), *EGFR* (R = −0.390, P < 0.001) (Fig. 5e) and *MKI67* (R = −0.376, P < 0.001) (Fig. 5f), but positively associated with the mRNA expression of *FOXA1* (R = 0.608, P < 0.001) (Fig. 5d). Conversely, *CD44* was found to be positively correlated with *FN1* (R = 0.253, P < 0.001) (Fig. 5g), *VIM* (R = 0.237, P < 0.001) (Fig. 5h), *YBX1* (R = 0.446, P < 0.001) (Fig. 5i), *EGFR* (R = 0.336, P < 0.001) (Fig. 5k) and *MKI67* (R = 0.215, P < 0.001) (Fig. 5l), while there was a significantly negative association between *CD44* and *FOXA1* (R = −0.402, P < 0.001) (Fig. 5j).

**Figure 6:**
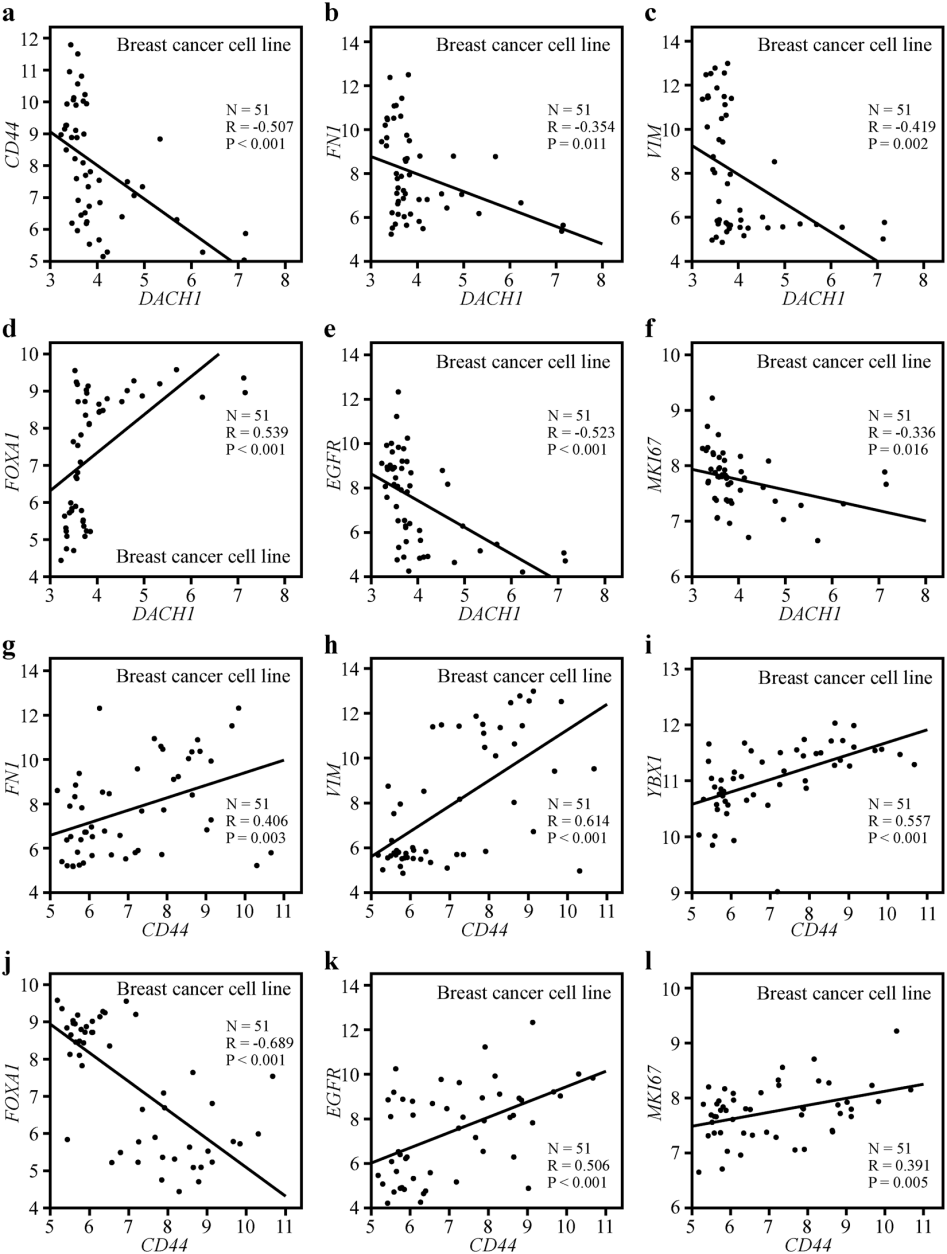
The association between the expression of *DACH1* and *CD44* with *VIM*, *FN1*, *YBX1*, *FOXA1*, *EGFR* and *MKI67* in breast cancer cell lines. Correlation analysis of data with a total of 51 breast cancer cell lines showed that *DACH1* was inversely correlated with *CD44* (**a**), *FN1* (**b**), *VIM* (**c**), *EGFR* (**e**) and *MKI67* (**f**), while positively associated with *FOXA1* (**d**). In contrast, *CD44* was parallel with *FN1* (**g**), *VIM* (**h**), *YBX1* (**i**), *EGFR* (**k**) and *MKI67* (**l**), but negatively related to *FOXA1* (**j**).

Breast cancer cell line data reported by Neve RM^25^, including a total of 51 different breast cancer lines from luminal-type (N = 25) or basal-like (N = 26), were also employed to evaluate the correlation between the expression of *DACH1* and *CD44* and the levels of *FN1*, *VIM*, *YBX1*, *FOXA1*, *EGFR* and *MKI67*. The results displayed that *DACH1* mRNA expression was inversely correlated with *CD44* (R = −0.507, P < 0.001) (Fig. 6a), *FN1* (R = −0.354, P = 0.011) (Fig. 6b), *VIM* (R = −0.419, P = 0.002) (Fig. 6c), *EGFR* (R = −0.523, P < 0.001) (Fig. 6e) and *MKI67* (R = −0.336, P = 0.016) (Fig. 6f) but positively associated with the mRNA level of *FOXA1* (R = 0.539, P < 0.001) (Fig. 6d), which were consistent with the results got in GSE20685. In contrast, *CD44* was parallel with *FN1* (R = 0.406, P = 0.003) (Fig. 6g), *VIM* (R = 0.614, P < 0.001) (Fig. 6h), *YBX1* (R = 0.557, P < 0.001) (Fig. 6i), *EGFR* (R = 0.506, P < 0.001) (Fig. 6k) and *MKI67* (R = 0.391, P = 0.005) (Fig. 6l) but negatively associated with *FOXA1* (R = −0.689, P < 0.001) (Fig. 6j), supporting the conclusion from the correlation analysis of human breast tumor samples (GSE20685).

### The opposite roles of *DACH1* and *CD44* in clinical outcomes of breast cancer patients

**Figure 7:**
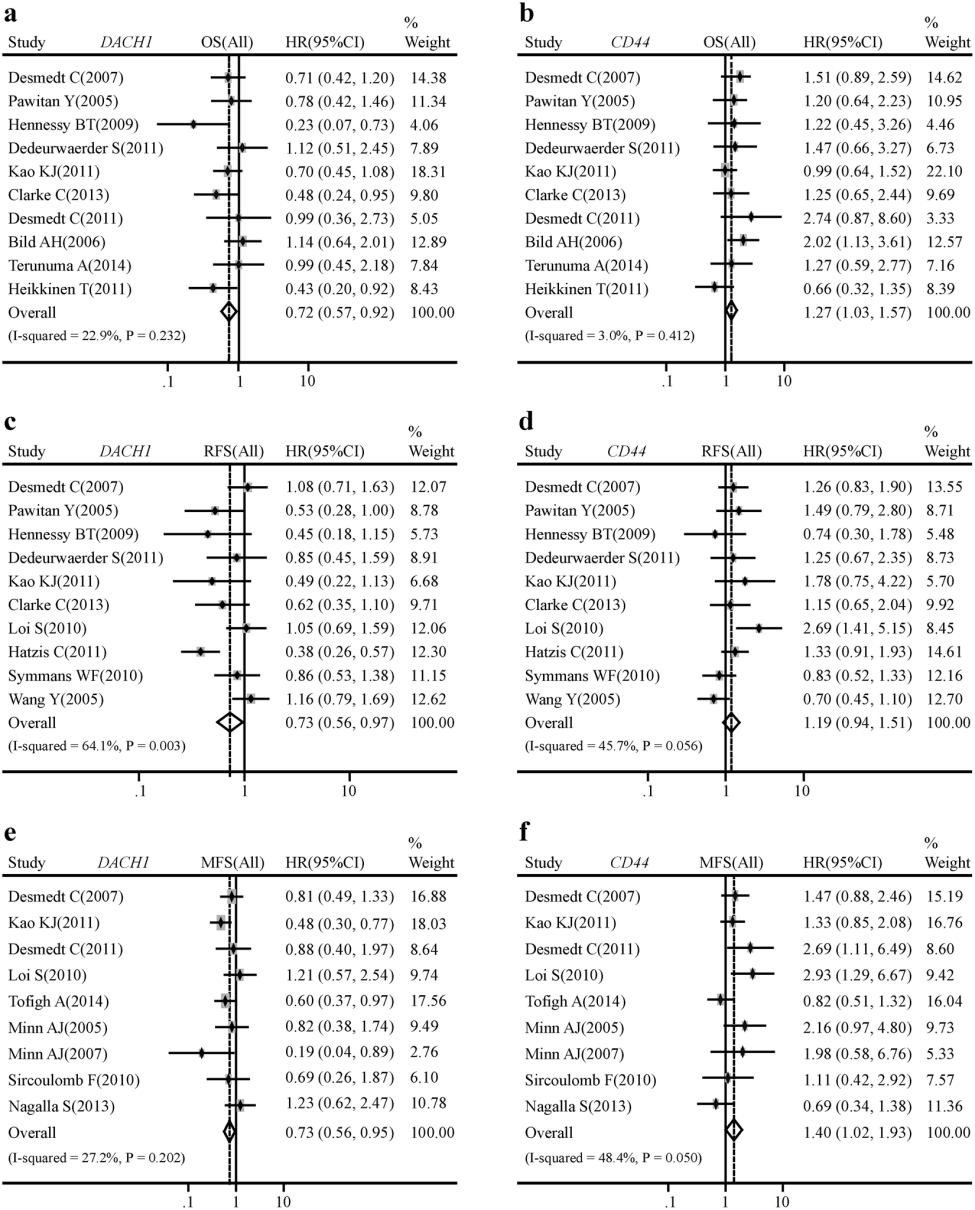
*DACH1* and *CD44* were related to clinical outcomes of breast cancer patients. Meta-analysis of a total of 19 public databases showed that breast cancer patients with higher *DACH1* mRNA level tended to have better overall survival (**a**), relapse-free survival (**c**), metastasis-free survival (**e**). On the contrary, patients with higher *CD44* mRNA expression had shorter time to death (**b**) and metastasis (**f**), but did not acquire statistically significant worse relapse-free survival in comparison to patients with comparatively lower *CD44* expression (**d**).

In order to assess the prognostic value of *DACH1* and *CD44* in breast cancer, a meta-analysis enrolling a total of 19 published Gene Expression Omnibus (GEO) databases and including 3574 breast cancer patients was performed^26–44^. The characteristics of these 19 GSE databases were displayed in Table 1. The results indicated that patients with higher mRNA expression of *DACH1* tended to enjoy longer time to death (HR: 0.72 (0.57–0.92), I^2^ = 22.9%, P = 0.232) (Fig. 7a), relapse (HR: 0.73 (0.56–0.97), I^2^ = 64.1%, P = 0.003) (Fig. 7c) and metastasis (HR: 0.73 (0.56–0.95), I^2^ = 27.2%, P = 0.202) (Fig. 7e). On the contrary, higher mRNA expression of *CD44* was directly related to worse OS (HR: 1.27 (1.03–1.57), I^2^ = 3.0%, P = 0.412) (Fig. 7b) and MFS (HR: 1.40 (1.02–1.93), I^2^ = 48.4%, P = 0.050) (Fig. 7f), but did not insignificantly contribute to worse RFS (HR: 1.19 (0.94–1.51), I^2^ = 45.7%, P = 0.056) (Fig. 7d). Our results demonstrated that DACH1 was a promising biomarker predictive of better clinical outcomes, while CD44 was an adverse factor for the survival performance of breast cancer patients.

**Table 1:** Characteristics of the included public microarray datasets in the meta-analysis.

First Author	GSE accession	Year	Duration (Months)	Patient Number	Detection	Platform
Desmedt C^26^	GSE7390	2007	163.2	198	Microarray	GPL96
Pawitan Y^27^	GSE1456	2005	102	159	Microarray	GPL96
Hennessy BT^28^	GSE10885	2009	106	89	Microarray	GPL887
Dedeurwaerder S^29^	GSE20711	2011	169	88	Microarray	GPL570
Kao KJ^30^	GSE20685	2011	156	327	Microarray	GPL570
Clarke C^31^	GSE42568	2013	100.9	104	Microarray	GPL570
Desmedt C^32^	GSE16446	2011	60	120	Microarray	GPL570
Loi S^33^	GSE6532	2010	176.8	327	Microarray	GPL96
Bild AH^34^	GSE3143	2006	156	158	Microarray	GPL8300
Terunuma A^35^	GSE39004	2014	120	61	Microarray	GPL6244
Heikkinen T^36^	GSE24450	2011	120	183	Microarray	GPL6947
Hatzis C^37^	GSE25066	2011	120	508	Microarray	GPL96
Symmans WF^38^	GSE17705	2010	196	298	Microarray	GPL96
Wang Y^39^	GSE2034	2005	180	286	Microarray	GPL96
Tofigh A^40^	GSE58644	2014	145	321	Microarray	GPL6244
Minn AJ^41^	GSE2603	2005	130	99	Microarray	GPL96
Minn AJ^42^	GSE5327	2007	156	58	Microarray	GPL96
Sircoulomb F^43^	GSE17907	2010	112	51	Microarray	GPL570
Nagalla S^44^	GSE45255	2013	127.4	139	Microarray	GPL96

## Discussion

DACH1, as an important member of RDGN, is widely expressed in epithelial cells and plays critical roles in normal organ development^45, 46^. However, its absent or lower expression contributes to the initiation and progression of various tumor types, including lung adenocarcinoma^14^, pancreatic cancer^47^, breast cancer^10^ and gastric cancer^48^. This study provided results that expression types of DACH1 and CD44 were opposite in breast cancer. Overexpression of DACH1 reduced the levels of CSC and EMT markers in breast cancer cell line and potently inhibited the ability of tumorgenesis in xenograft model. Besides, correlation analysis exhibited that *DACH1* and *CD44* behaved completely differently in the correlations with *FN1*, *VIM*, *YBX1*, *FOXA1*, *EGFR* and *MKI67*. Importantly, *DACH1* serves as a protective factor, while *CD44* is an unfavorable element for the prognosis of breast cancer patients. Thus, we concluded that DACH1 might exert inhibitory effects on the development of breast cancer partly by suppression of EMT inducers and CSCs markers, especially CD44.

Carcinogenesis may derive from the acquisition of a plethora of oncogenic mutations, sequential suppression of endogenous growth inhibitors^49^ and the loss control over pivotal cellular functions^50^. DACH1 accounts for the carcinogenesis of various tumor types, including human breast cancer^51^. Previous studies have implicated that DACH1 negatively regulated cellular proliferation and tumor growth by repressing cell cycle protein cyclin D1 in both breast cancer^11^ and renal clear cell cancer^16^. Restoration of DACH1 suppressed clone formation of renal clear cell cancer cells *in vitro* as well as tumor growth *in vivo* through the inhibition of cyclin D1 transcription via associating with AP-1 protein^16^. Furthermore, hypermethylation of DACH1 promoter region itself led to carcinogenesis and it also exerted inhibitory effects on tumor initiation through activating transforming growth factor-beta (TGF-beta) signaling^15^. Besides, DACH1 inhibited cellular growth in an NAD and p53-dependent manner by direct protein-protein association in breast cancer^52^. On the contrary, CD44, a family of transmembrane glycoproteins, exerted promoting roles in tumorigenesis of various cancer types^20^. Up-regulation of the cleaved intracellular domain of CD44 (CD44ICD) enriched the mammosphere formation, while the blockage of CD44ICD reversed the effects^53^. Immunohistochemistry analysis on human breast cancer tissues and expression analysis of GSE42568 indicated that CD44 remarkably increased in breast carcinoma in comparison with normal breast tissues^22^. CD44 functions in carcinogenesis through binding to extracellular matrix components and messenger molecules in tumor environment^54^.

Several lines of evidence indicated that DACH1 expression correlated with tumor differentiation. Immunohistochemistry analysis of clear cell renal cancer tissues displayed that DACH1 was inversely correlated with tumor grade^16^. About 60% of cells in low-grade tumors expressed DACH1, and less than 20% cells in grade III tumors expressed DACH1^16^. Similar phenomenon was also found in breast cancer^13^. DACH1 was remarkably enriched in low-grade breast tumors^13^. In contrast, CD44 positively correlated with breast tumor grade^21, 22^. Our previous study displayed a positive association between CD44 expression and breast tumor grade at both mRNA and protein levels^21, 22^. This paper further supported this opposite tendency of DACH1 and CD44 in low-grade and high-grade breast carcinoma, respectively.

Majorly according to the status of ER, PR and Her2, breast cancer is grouped into four distinct subtypes including luminal, Her2-positive, basal-type and normal-like. Among these, luminal tumor patients accounted for the most part of breast carcinoma population and have relatively good clinical outcomes, while patients with basal-like tumor endowed with malignancy features have significantly poor prognosis. Previous study has demonstrated that nuclear DACH1 is a biomarker of luminal breast cancer^13^. Breast tumors with positive ER–alpha and co-expressing PR-alpha were most likely to highly express DACH1, and nuclear DACH1 expression was positively correlated with luminal marker FOXA1 and inversely associated with basal-like markers EGFR^13^. In contrast, our previous meta-analysis showed that *CD44* mRNA was remarkably enriched in basal-like breast cancer compared with luminal-type breast tumor^21^. *CD44* was also positively correlated with basal markers *EGFR*, *KRT5* and *KRT17*, and inversely associated with luminal marker *FOXA1* ^22^. Our results supported that luminal breast neoplasm tended to be with high DACH1 expression and low CD44 level, while basal-like tumors were most likely to be the inverse type. The correlation analysis further indicated that DACH1 was significantly inversely associated with CD44.

CSCs were composed of a subset of tumor cells with the expression of stem cell-associated markers and enhanced capacity for tumorigenesis, metastasis and therapy-resistance^55^. It has been implicated that endogenous DACH1 participated in the negative regulation of CSCs^18^. DACH1 suppressed the expression of stem cell markers SOX2, Nanog and KLF4 through binding to the promoters of these genes^18^. On the contrary, CD44, as a well-known CSCs marker, positively monitored the levels of CSCs markers^20^. Nuclear location of cleaved CD44 intracellular domain transcriptionally activated stemness factors Nanog, Sox2 and Oct4^53^.

EMT is a complex and highly conserved process which enhances cellular invasiveness, being critical for metastasis of various solid tumors and considered to be promising therapeutic targets^56^. EMT was dysregulated by a complex network during tumor development. Knock-down of DACH1 in breast cancer cells MCF-7 and T47D promoted the morphology change from epithelial phenotype to mesenchymal pattern and interfered with cell–cell contact, accompanied by down-regulation of epithelial marker E-cadherin, resulting in cell migration and invasion^10^. DACH1 also transcriptionally suppressed the activity of Snail, leading to the activation of E-cadherin in breast cancer cells^10^, but the complex of DACH1 and Snail could bind to the E-box of E-cadherin promoter then contributing to the reduction of E-cadherin^10^. In addition, DACH1 reduced the expression of the mesenchymal marker Snail through suppressing the activity of the Y box-binding protein, an important EMT inducer^9^. Besides, previous study has revealed that DACH1 decreased both in breast cancer cell lines and tissues accompanied by relatively high proportion of CSCs^18^. Inversely, CD44 not only promoted EMT, but also was upregulated by some mesenchymal markers^20^. Previous studies have demonstrated that mesenchymal genes including *ZEB1*, *TWIST1*, *SNAI1* and *SLUG* were positively correlated with CD44 expression^20^. The switch from CD44 variant isoforms to its standard isoform is essential to undergo EMT for normal epithelial cells^57^.

DACH1 played important roles in invasion and migration of various neoplasms, such as lung adenocarcinoma^14^, gastric cancer^48^, pancreatic cancer^47^ and breast cancer^12^. Several lines of evidence showed that DACH1 is absent or suppressed in poor prognosis breast cancer^58^. Breast cancer patients with reduced DACH1 expression had three years shorter time to death in comparison to those with normal DACH1 levels^11^. Immunohistochemistry analysis showed that higher nuclear level of DACH1 was predictive of longer disease-free interval, cancer relapse-free survival and distant metastasis-free survival over 5 years post diagnosis^13^. In consistence, our analysis of GSE databases showed that higher mRNA level of DACH1 was parallel with better OS, RFS and MFS. The protected effect of DACH1 in the prognosis of breast cancer patients could be explained partly by negatively interplaying CSCs and EMT^10, 59^. Besides, DACH1 inhibited breast cancer migration and invasion also via suppressing oncogene function through targeting interleukin-8^12^. Altogether, these studies suggested that DACH1 could be a valuable molecular marker for prognosis, thereby detection of DACH1 level is useful for therapeutic stratification of breast cancer patients. In contrast, CD44 contributed to tumor formation and progression predictive of poor clinical outcome^20^. CD44 functioned as an oncoprotein and knockdown of CD44 remarkably attenuated the migration and invasion of breast cancer cells MDA-MB-231 and Hs578T by modulating c-Src transcription^60^. According to the analysis of 448 primary breast tumors, CD44 was parallel with enhancive distant recurrence and decreased disease-free survival^61^.

In this study, 19 public datasets were enrolled to assess the correlation between the mRNA levels of *DACH1* and *CD44* and the survival performance of breast cancer patients. However, there are still some limitations: 1) the overall sample size is limited. Some data were not available when the meta-analysis was conducted; 2) the platforms used to evaluate the mRNA expression of *DACH1* and *CD44* are different; 3) the results at the mRNA levels might not be consistent with those at protein abundance because there is a complex regulation network from mRNA to protein.

In conclusion, this study confirmed that DACH1 and CD44 inversely related in breast cancer, different grade tumors and different subtypes. DACH1 was negatively associated with CD44 *in vitro* and *vivo*. DACH1 served as a good prognostic marker, and CD44 was an unfavorable element of breast cancer patients. Thus, we concluded that CD44 might be a novel target of DACH1.

## Materials and Methods

### Immunohistochemical staining

To evaluate the expression of DACH1 and CD44 in normal breast versus breast tumor tissues, grade 1–2 versus grade 3 tissues as well as luminal-type and basal-like human breast carcinoma tissues, two commercially available tissue microarray (TMA) slides (BR1502–97 and BR1502-98, US Biomax, Inc, Rockville, MD) containing histologically confirmed tissues were purchased for immunohistochemistry (IHC) analysis. In addition, IHC was also employed to compare the expression of CD44, Fibronectin, Vimentin, Sox2, Myc, EGFR and Ki-67 in Met-1 nude mice xenograft tumors with overexpression of DACH1 and the controls. The Specific primary antibodies against DACH1 (10914-1-AP, ProteinTech Group), CD44 (15675-1-AP, ProteinTech Group), Fibronectin (sc-8422, Santa Cruze), Vimentin (5741, Cell Signaling Technology), Sox2 (AB5603, Millipore), Myc (sc-40, Santa Cruze), EGFR (sc-03, Santa Cruze) and Ki-67 (ab15680, Abcam) were utilized for IHC with a 2-step protocal^62^.

### Analysis and quantification of staining

For quantification, a total of six 200× magnifications of each kind of protein were selected for IHC scoring. Two experienced pathologists assessed the immunohistochemical score independently. Scores were calculated on intensity and proportion of positive staining tumor cells in the whole tissue stains according to the Fromowitz Standard as described above^63^. The staining intensity was scored as 0 (no staining), 1 (weak staining, light yellow), 2 (moderate staining, yellow brown) and 3 (strong staining, brown). The proportions of stained tumor cells were classified as 1 (0–25% positive cells), 2 (26–50% positive cells), 2 (51–75% positive cells) and 3 (76–100% positive cells). The multiplication for intensity and proportion was utilized to represent the protein levels of DACH1, CD44, Myc, Sox2, Fibronectin, Vimentin, EGFR and Ki-67.

### Cell culture

The breast cancer cell line Met-1, was cultured in high-glucose Dulbecco’s modified Eagle’s medium (DMEM) supplemented with 10% fetal bovine serum (FBS, Life Technologies, Inc.). All cells were grown 37 °C in a humidified incubator with 5% CO_2_.

### Western blot

Cells were washed with cold PBS, scraped into RIPA buffer and centrifuged. The cell lysates were subjected to 10% SDS-PAGE and transferred to a polyvinylidene fluoride (PVDF) hybridization transfer membrane. The primary antibodies used were as follows: HA (Sc-7392, Santa Cruze), CD44 (15675-1-AP, ProteinTech group), Fibronectin (sc-8422, Santa Cruze), Vimentin (5741, Cell Signaling Technology), p21cip1 (sc-6246, Santa Cruze), p27kip2 (sc-1641, Santa Cruze) and β-actin (Sc-47778, Santa Cruze). Secondary staining and detection were carried out in accordance with standard protocols^16, 64^.

### RNA profiling by microarray

DNA-free total RNA isolated from Met-1 cells expressing GFP or *DACH1* were used to probe Affymetrix Gene 1.0 arrays (Affymetrix, Santa Clara, CA). RNA quality was determined by gel electrophoresis. Analysis of the arrays was performed using GeneSpring. Arrays were normalized using robust multi-array analysis, and the *p* value of 0.05 was applied as a statistical criterion for differentially expressed genes.

### Meta-analysis for DACH1 on published Gene Expression Omnibus (GEO) databases

We carried out a comprehensive search of relevant GEO databases for mRNA expression of *DACH1* and *CD44* through ArrayExpress and Oncomine. The datasets meeting the following criteria were included: 1) the datasets were about human breast cancer; 2) the mRNA expression of *DACH1* and *CD44* was measured in these databases; 3) clinical outcomes of patients were showed in these databases; 4) the sample capacity was more than 50. Only the latest and most complete datasets were included when several databases shared common patients. At last, a total of 19 independent human breast cancer microarray databases with the mRNA expression of *DACH1* and *CD44* and required the survival information of breast cancer patients were enrolled in this systematic analysis.

Cutoff value for *DACH1* and *CD44* was median expression. OS, RFS and MFS were evaluated by Cox proportional hazard ratio (HR) and 95% confidence interval (95% CI). HRs was employed to assess the survival outcome of breast cancer patients with high mRNA expression of *DACH1* and *CD44* and HR >1 indicated that high expression of *DACH1* and *CD44* predicted worse survival of patients. The random-effect model was employed when heterogeneity was present, and the fixed-effect model was used when homogeneity was demonstrated. Heterogeneity of publication was evaluated by means of the Chi-square-based-Q statistic and inconsistency index (I^2^) statistic. Statistical analysis was performed based on the guidelines of Meta-Analysis of Observational Studies. The STATA software package (version 12.0) (Stata Corp LP, College Station, TX, USA) was employed to perform the meta-analysis.

### Analysis of gene expression data

GSE20711, available through GEO databases and containing 45 luminal-type and 22 basal-like breast carcinoma cases, was analyzed to evaluate the mRNA expression of *DACH1* and *CD44* in basal-like carcinoma in comparison with that in luminal-type tumors.

GSE20685, containing a total of 327 patients of breast cancer, was interrogated to assess the correlation between *DACH1* and *CD44*, *FN1*, *VIM*, *FOXA1*, *EGFR*, *MKI67* as well as the correlation between *CD44* and *FN1*, *VIM*, *YBX1, FOXA1*, *EGFR and MKI67* in breast cancer tissues.

Breast cancer cell line data of 51 breast cancer cell lines of different molecular subtypes published by Neve RM, *et al*.^25^ were also employed to evaluate whether the correlation in breast cancer cells was consistent with that in breast tumor tissues.

### Nude Mice Study

The animal protocols were approved by the ethics committee of the Tongji Hospital of Huazhong University of Science and Technology. The methods used in this section were in accordance with the relevant guidelines and regulations. 1 × 10^5^ Met-1 cells expressing GFP control or DACH1 were implanted subcutaneously into 4–6-week-old athymic female nude mice purchased from Wuhan Hamilton Biological Polytron Technologies Inc. The tumor growth was measured twice weekly for 3 weeks by using a digital caliper. Tumor weight was measured when mice were sacrificed on day 27 after cells implantation.

### Statistical analysis

Correlation analyses were performed using SPSS 20 statistical software (SPSS Inc., Chicago, IL, USA). The Student’s t-test was applied to evaluate the differences in groups as appropriate and the significance level was set at 0.05. The correlation analysis was evaluated by a χ^2^- test. A two-tailed p value < 0.05 was considered statistically significant.
